# Inter-Individual Variation in Anti-Parasitic Egg Rejection Behavior: A Test of the Maternal Investment Hypothesis

**DOI:** 10.1093/iob/obaa014

**Published:** 2020-05-06

**Authors:** M E Hauber, M Abolins-Abols, C R Kim, R T Paitz

**Affiliations:** 1 Department of Evolution, Ecology, and Behavior, School of Integrative Biology, University of Illinois at Urbana-Champaign, Urbana, IL 61801, USA; 2 Department of Biology, University of Louisville, Louisville, KY 40292, USA; 3 School of Biological Sciences, Illinois State University, Normal, IL 61790, USA

## Abstract

Hosts of avian brood parasites may reduce or forego the costs of caring for foreign young by rejecting parasitic eggs from the nest. Yet, many host species accept parasitic eggs and, even among rejecter species, some individuals go on to incubate and hatch them. The factors explaining the variation in egg rejection between species have received much theoretical and empirical attention, but the causes of intraspecific variation in different individuals’ propensity for accepting parasitic eggs are less well understood. Here we tested the maternal investment hypothesis, which predicts that hosts with costlier clutches will be more likely to reject parasitic eggs from their nest. We studied variation in the egg rejection responses of American robins (*Turdus migratorius*), a robust egg-rejecter host of the brood parasitic brown-headed cowbird (*Molothrus ater*), to 3D-printed cowbird-sized eggs which were painted dark blue, a color known to induce variable and repeatable egg rejection responses in individual robins. Costlier clutch investment was estimated by earlier laying date, larger clutch size, heavier unincubated yolk mass, and variable yolk steroid hormone concentrations. There was no statistical support for most of our predictions. However, we detected more concentrated and greater overall amount of deoxycorticosterone deposited in egg yolks of rejecters relative to acceptors, although this accounted for no more than 14% of variance in the data. Future work should test experimentally the potential physiological linkage between maternal egg yolk steroid investment and egg rejection propensity in this and other host species of avian brood parasites.

## Synopsis

**Egyedek közti variáció az antiparazitikus tojás-diszkriminációban: az anyai befektetés hipotézis tesztelése**

**Kivonat**

A költésparazita madárfajok gazdái csökkenthetik vagy megszüntethetik az idegen fióka nevelésének költségeit úgy, hogy eltávolítják a fészekből a paraziták tojásait. Ennek ellenére több gazdafaj is elfogadja a parazita tojásokat, sőt az elutasító gazdafajok körében is akadnak olyan egyedek, amelyek elfogadják és kikeltik az idegen tojást. Bár az eddigi elméleti és kísérleti kutatások nagy hangsúlyt fektettek a különböző gazdafajok közti, költésparazita tojások diszkriminacióját befolyásoló tényezők feltárására, a parazita tojások elfogadásának gazdafajon belüli varianciája kevésbé tisztázott. Jelen kutatásunkban az anyai befektetés hipotézisét vizsgáltuk, melynek értelmében azok az egyedek, amelyek több energiát fektetnek fészekaljukba, nagyobb valószínűséggel távolítják el a költésparazita tojásait a fészekből. A vándorrigónak (*Turdus migratorius*) a parazita tojást elutasító viselkedését vizsgáltuk meg a költésparazita barnafejű gulyajáró (*Molothrus ater*) tojásához hasonló méretű, de sötétkék színű, 3 D nyomtatóval készített műtojásokat használva. A költési periódusban korábban letojt, több tojásból álló, nagyobb inkubálatlan tömegű és változatos szteroid hormonkoncentrácijú sárga szíkű tojásokkal rendelkező fészekaljakat tekintettük a magasabb anyai befektetésű fészekaljaknak. Feltevéseink nagyrészét statisztikai eredményeink nem támasztották alá. Mindezek ellenére, a parazita tojásokat elfogadó egyedekéhez képest, az idegen tojást elutasító egyedek tojásai sárga szíkanyagában nagyobb koncentrációban és mennyiségben volt jelen a dezoxikortikoszteron, bár ez az adatainkra jellemző varianciának csupán 14%-át magyarázta. További kísérletes vizsgálatok szükségesek ahhoz, hogy feltárhassuk az esetleges fiziológiás kapcsolatot az anyai tojas szteroid hormone befektetés és a parazita tojások elutasításának gyakorisága között ennél a gazdafajnál, és más költésparazita gazdáknál egyaránt. *Translated kindly by Attila Marton, Debrecen University.*

## Introduction

In response to costly parasitism, hosts may forego some or all fitness losses by resisting or rejecting parasitic individuals (Aviles 2018). Many hosts of avian obligate brood parasites, for example, attempt to prevent parasitism of the breeding attempt altogether by front-loading defenses through hiding their nests and mobbing the parasites (Feeney et al. 2014). In turn, in other host species, the rejection of foreign egg(s) in the nest is a widespread and effective means to reduce the costs of brood parasitism (Rothstein 1975; Manna et al. 2017). Yet, some individuals, even in predominantly egg-rejecter species, accept the parasitic egg, and go on to incubate it and raise the foreign chick (Lowther 1981). The factors underlying variation in egg rejection behaviors between potential host species have received much theoretical and empirical attention (reviewed in Davies 2000;Soler 2017). What explains inter-individual level variation in egg rejection behavior intraspecifically remains one of the major open questions in avian host-brood parasite interactions (Abolins-Abols and Hauber 2018).

For example, American robins (*Turdus migratorius*, hereafter robins) serve as occasional hosts to the obligate brood parasitic brown-headed cowbird (*Molothrus ater*; hereafter cowbird) throughout North America (Rothstein 1982). Nearly all robins reject natural and experimental parasitism by cowbirds (Friedmann 1929; Rasmussen et al. 2009; Luro et al. 2018), whereas anecdotal reports exist of acceptor robins that also go on to hatching and provisioning the cowbird chick (Lowther 1981 and references therein; M. Abolins-Abols and M. E. Hauber, personal observations). Critically, the perceived threshold to accept versus reject foreign eggs by robins lies along the blue-beige-brown gradient of natural avian egg-color diversity (Croston and Hauber 2014a; Hanley et al. 2017), such that some bluish model eggs are rejected at intermediate rates at the population level, but either consistently accepted or consistently rejected by the same robins (e.g., Croston and Hauber 2014b; Luro and Hauber 2017). This allows the use of painted model cowbird eggs to induce and analyze correlates of inter-individual variation in the robins’ intermediate egg rejection responses to artificial brood parasitism (Abolins-Abols and Hauber 2020).

When egg rejection is inter-individually variable, female hosts may balance some of the costs incurred during egg rejection (e.g., cognitive, temporal, and energetic costs of clutch assessment, mistakenly removing or accidentally damaging own eggs) against the benefits gained from the investment made into a given breeding attempt (e.g., Hauber et al. 2019). Evolutionary cost–benefit theory then predicts that the greater the maternal investment into the current clutch, the greater the probability that the foreign egg is rejected to reduce parasitism-incurred costs in the nest (Reeve 1989; Davis et al. 1996; Moskat and Hauber 2007). Here we tested predictions of this “maternal investment” hypothesis by inducing variable egg rejection in wild robins and correlating an individual’s response behavior with proxy metrics of natural levels of maternal investment into the current breeding attempt. Specifically, based on the published literature, we considered greater maternal investment as earlier laying date (Pilz et al. 2003), larger clutch size (Petrie and Williams 1993), and heavier unincubated yolk mass (Uller et al. 2005), whereas the literature had no directional prediction about the maternal costs or benefits of variable yolk steroid hormone concentrations (Kristofik et al. 2014; Bowers et al. 2016).

In previous experiments with foreign egg colors, mistaken rejection of the robin’s own eggs was not observed (e.g., Croston and Hauber 2015a; Luro et al. 2018), except in one study where the foreign egg’s color was transient (using thermochromic paint: Hauber et al. 2019). In turn, experimentally parasitized robins pay a significant (−15% fledging success relative to non-parasitized nests) cost for raising a cowbird chick alongside their own nestlings (Croston and Hauber 2015b). Thus, our specific prediction is that maternally costly investment should favor the rejection of the foreign model egg from the robin’s nest.

Some species-level comparisons of relative avian brood parasite versus host investments into egg yolk-deposited nutrients, antioxidants, and hormones are already available (e.g., Hauber and Pilz 2003; Hahn et al. 2005; Schwabl et al. 2007; Hargitai et al. 2010; Royle et al. 2011; Igic et al. 2015). In contrast, assaying for physiologically-informed clutch-investment metrics of individual host females remains a much understudied integrative dimension of the otherwise extensive analyses of egg rejection behaviors in avian host–brood parasite interactions (but see Hahn et al. 2017).

## Methods

### Field work

This research was approved by a research protocol at the University of Illinois at Urbana-Champaign (IACUC: #17259) and by permits from US Federal (Fish and Wildlife Service: #MB08861A-3) and Illinois State agencies (Department of Natural Resources: #NH19.6279).

We studied the egg rejection behaviors of free-living American robins at two nearby (∼8 km apart) tree farms in Champaign County, IL, USA, during the 2019 breeding season (May–June). Extensive details of the study area and methods are given in Luro and Hauber (2017) and Scharf et al. (2019). In brief, nests were located by visually searching for the bulky nest structure of robins; when located, the contents were examined using a telescopic mirror. Because maternal investment into egg yolks, including yolk hormones, can vary with laying order (e.g., Schwabl 1993, but see Kumar et al. 2018), we aimed to standardize our study by only using nests where the identity of the first laid egg was known for collection and yolk analyses (see below); therefore, we monitored several mid-construction and completed but empty nests prior to the first egg appearing. We did not catch and band robins for this investigation; however, to reduce pseudo-replication due to the same breeding robin(s), when possible we conducted our work in bouts, whereby treatment was initiated in simultaneously active nests within a 1-week period in one study site, then we moved to the second site and assessed the next set of active nests within another 1-week long period before returning to the first site.

When a nest contained a single robin egg (Day 0), we marked that egg with a black felt-tip permanent marker Sharpie Pen^™^, and added a deep-blue painted, 3D-printed, and brown-headed cowbird-egg shaped, sized, and weighted model egg (model “Cow bird egg smooth” at Shapeways.com in white nylon; painted by us with a non-toxic Winsor and Newton Galeria Ultramarine Blue^©^ acrylic paint in three coats; for more information, see Luro and Hauber 2017; for the reflectance spectrum, see Figure 2 “blue” in Croston and Hauber 2014a). We used this model egg color and size because it is known to be rejected both at intermediate (30–70%) rates (Luro and Hauber 2017; Abolins-Abols and Hauber 2020; this study) and in a repeatable fashion by individual robins both in our study area (Luro and Hauber 2017) and elsewhere in North America (Croston and Hauber 2014b).

We returned to the nest the next day (Day 1), inspected nest content, removed the first laid robin egg (already marked), and replaced it with an unincubated natural robin egg collected from abandoned local robin nests to maintain the number of natural robin eggs in the clutch. In *Turdus* thrushes, replacing a natural egg or adding to a clutch does not alter foreign egg rejection rates (Grim et al. 2011). This timing of the natural egg’s removal was set because robins in our population have a typical clutch size of three to four eggs (see “Results” section) and our aim was to obtain unincubated first-laid eggs for the egg yolk analysis. We then revisited the nest daily for four additional consecutive days (up to Day 5) to establish natural clutch size and the presence of the model egg.

We assumed that robins lay a single egg per day (Rowe and Weatherhead 2009) and that a clutch was completed when the number of robin eggs did not change across two or more consecutive days. At a subset of nests (6/28), we found nests at the two-egg stage already; for these nests, we marked both of the eggs with the felt tip pen, added the model egg, and randomly removed and replaced one of the first two robin eggs. Excluding these nests and eggs from the analyses did not alter our conclusions statistically.

The mean, median, and modal clutch size in our study sample were four eggs and over 90% of robins in Illinois initiate incubation by the first day after clutch completion (Rowe and Weatherhead 2009), which translates into Day 4 in our study. We, therefore, assessed egg rejection behavior on Day 4 following the onset of egg insertion; this assured the exposure of most robins in our study to the model egg upon their respective onsets of incubation.

Nest desertion is not a response to experimental parasitism in robins (Croston and Hauber 2014a), and both deserted (cold eggs of constant clutch size on consecutive days) and depredated (all eggs missing or broken robin eggshells) were removed from the analyzed dataset (hence, accounting for variation in the sample size across the different days of monitoring, see “Results” section). We also did not visit one nest on Day 2 due to a logistical constraint but resumed its monitoring on Day 3 onwards.

Note that our study induced and monitored variation in robins’ egg rejection responses not as a function of different treatments, but rather as an outcome of the robins’ own responses to a single model-egg type. Thus, this is not a controlled experimental study with treatments and controls, and instead should be interpreted as a correlational study, despite the implementation of artificial parasitism.

### Yolk analysis

Eggs were transported to the lab at ambient temperatures, wrapped whole in aluminum foil, and stored at −20°C until steroid extraction (median duration of storage: 22 days, range 3–40), which generally followed the methods of Merrill et al. (2019). In brief, steroids were extracted from yolks using solid phase extraction. To do this, eggs were thawed, and the yolk was physically separated from the albumen and weighed in total before being homogenized. A 0.5 g aliquot of yolk was then transferred to a 15 mL conical vial and 4 mL of 100% methanol was added to each. The mixtures were then vortexed and placed at −20°C overnight to precipitate neutral lipids. Samples were then centrifuged for 20 min at 2000 rpm and the supernatant (∼4 mL) was transferred to a 50 mL vial and diluted with 46 mL of nanopure water (Kozlowski et al. 2009).

To extract steroids from the diluted supernatant, samples were passed through C18 Sep-Pak cartridges (Waters, Ltd., Watford, UK) charged with 5 mL methanol and rinsed with 5 mL of nanopure water before the sample was run through the cartridge (Newman et al. 2008). Samples were run at a drip rate of ∼2 mL/min and steroids were eluted with 5 mL of diethyl ether and dried under nitrogen gas. Dried samples were then submitted to the Metabolomics Laboratory of the Roy J. Carver Biotechnology Center, University of Illinois at Urbana-Champaign, for the quantification using LC/MS/MS (*sensu*Merrill et al. 2017, 2019). We aimed to quantify 28 different steroids but were able to detect only 10 above the instrumentational threshold levels (which were ∼10 pg for all steroids), using the approach described in Merrill et al. (2019) who analyzed egg yolk steroids in seven species of Illinois birds, including robins.

We assessed two metrics for each hormone analysis: first, we used the hormone concentration measures from the yolk (i.e., hormone concentration); second, we calculated total hormone investment into the yolk by multiplying the yolk’s weight with each hormone’s respective concentration (i.e., hormone investment).

### Statistical analyses

The concentration and total hormone investment of some yolk hormones were strongly positively (most), negatively (some), or weakly (some) correlated with each other, therefore we used two principal components analyses (PCA) on the correlation matrices without imputation to reduce the dimensionality of these datasets. The first three principal component (PC) scores explained each over 10%, and cumulatively 71% and 69% of the variance for the hormone concentration and total hormone investment metrics, respectively (Tables 1 and 2), and these were used as our two maternal endocrine investment metrics in our initial statistical tests.

**Table 1:** PC eigenvectors for the concentrations of the 10 detected steroid hormones from egg yolks of American robins

Steroid	PC1	PC2	PC3
Estrone	0.10489	0.29649	0.00031
Androstenedione	0.30587	−0.40993	0.32449
Testosterone	0.17582	−0.55459	0.05444
DHEA	0.33259	−0.28895	0.40259
Etiocholanolone	0.24999	0.18784	0.27461
Progesterone	0.42428	0.06698	−0.26790
Pregnenolone	0.33454	0.37136	0.19229
17a-hydroxyprogesterone	0.41447	−0.05814	−0.38753
Deoxycorticosterone	0.40086	0.01272	−0.49960
Pregnanedione	0.25886	0.41445	0.38175
*Explained variance*	*39.4%*	*21.2%*	*10.4%*
*∑Explained variance*	***70.9%***		

**Table 2:** PC eigenvectors for the concentration 10 detected 10 steroid investments (concentration × total yolk mass) from eggs of American robins

Steroid	PC1	PC2	PC3
Estrone	0.04826	0.25327	−0.14405
Androstenedione	0.35604	−0.33610	0.32054
Testosterone	0.24367	−0.54324	0.08919
DHEA	0.36011	−0.20036	0.41810
Etiocholanolone	0.20059	0.18805	0.27751
Progesterone	0.42240	0.08700	−0.27092
Pregnenolone	0.27955	0.46485	0.15882
17a-hydroxyprogesterone	0.42429	−0.02599	−0.36765
Deoxycorticosterone	0.38739	0.01736	−0.50124
Pregnanedione	0.23443	0.47711	0.36289
Explained variance	*37.2%*	*19.7%*	*12.2%*
∑ Explained variance	***69.1%***		

After reducing the dimensionality of the hormone data, we analyzed if the endocrine and life history maternal investment metrics were related to each other using correlation analyses. We then used linear mixed models to test the impact of date on PC scores and individual hormone levels, with site as a random effect. Finally, we used generalized logistic mixed model analyses to test our predictions of how rejection of the deep-blue model egg (Day 4: accepted/rejected) was related to the metrics of maternal investment (date, clutch size, yolk mass, and hormone levels), with study site as a random effect.

All statistical tests were set with α ≤ 0.05 and conducted in JMP 12.0 (SAS Inc., Cary, NC, USA). The data are shared and available at Figshare’s doi: 10.6084/m9.figshare.12203186.

## Results

Cumulative rejection rates of the deep-blue model eggs gradually increased across the period of monitoring each nest, from 11% on Day 1 (*n* = 28 nests), through 46% on Day 2 (*n* = 26), 60% on Day 3 (*n* = 27), 70% on Day 4 (*n* = 27), to 80% on Day 5 (*n* = 25).

For the 10 steroids that were detectable in robin eggs, there were 4 progestogens (pregnenolone, progesterone, 17α-progesterone, and pregnanedione), 4 androgens (dehydroepiandrosterone [DHEA], androstenedione, testosterone, and etiocholanolone), 1 estrogen (estrone), and 1 mineralocorticoid (deoxycorticosterone). PC score loadings of individual hormones produced from steroid concentrations (Table 1) or absolute amounts (Table 2) were very similar and in the subsequent section, we focus on hormone concentrations only. Specifically, PC1 had positive loadings from all 10 detectable steroids (Table 1) and the strongest loading was that of progesterone with a loading of 0.42. For PC2, the androgens DHEA, androstenedione, and testosterone all loaded negatively while estrone, progesterone, and pregnanedione loaded positively (Table 1). Finally, PC3 had the strongest negative loading (−0.50) from the glucocorticoid precursor, deoxycorticosterone.

**Fig. 1:**
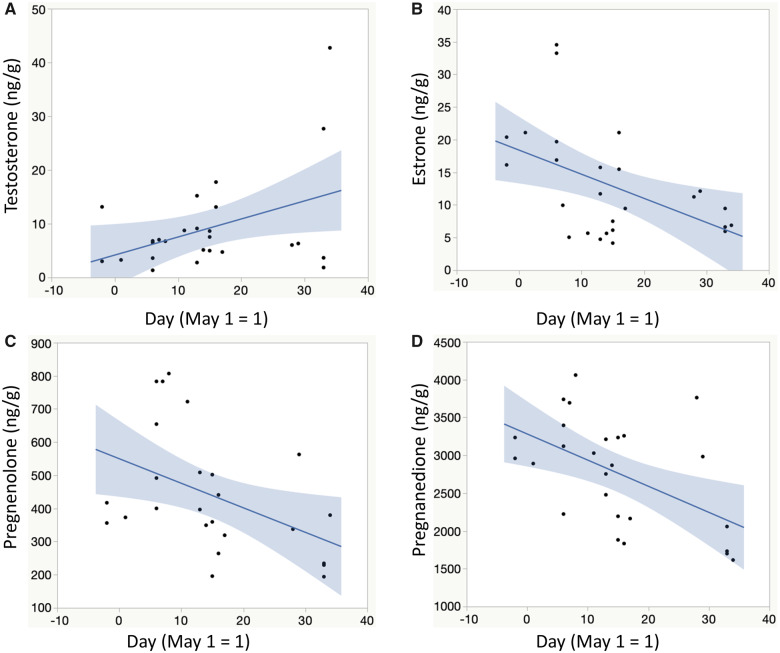
Seasonal shift in representative maternally-provisioned hormone concentrations in the yolk (ng hormone/g yolk) of unincubated first-laid eggs of American robins. The 95% confidence interval of the mean slope is indicated in the shaded areas.

None of the hormone concentration PCA score metrics were related to yolk mass or clutch size statistically (all *P* > 0.05) whereas PC1 and PC2 were both negatively related to clutch laying date (*R* ≤ −0.39, *P* ≤ 0.04; data not shown). *Post hoc* analyses revealed that yolk testosterone concentrations increased (*R* = 0.40, *P* = 0.04), whereas concentrations of yolk estrone, pregnenolone, and pregnanedione decreased (*R* ≤ −0.43, *P* ≤ 0.02) with the advancing breeding season (Figure 1A–D).

**Fig. 2:**
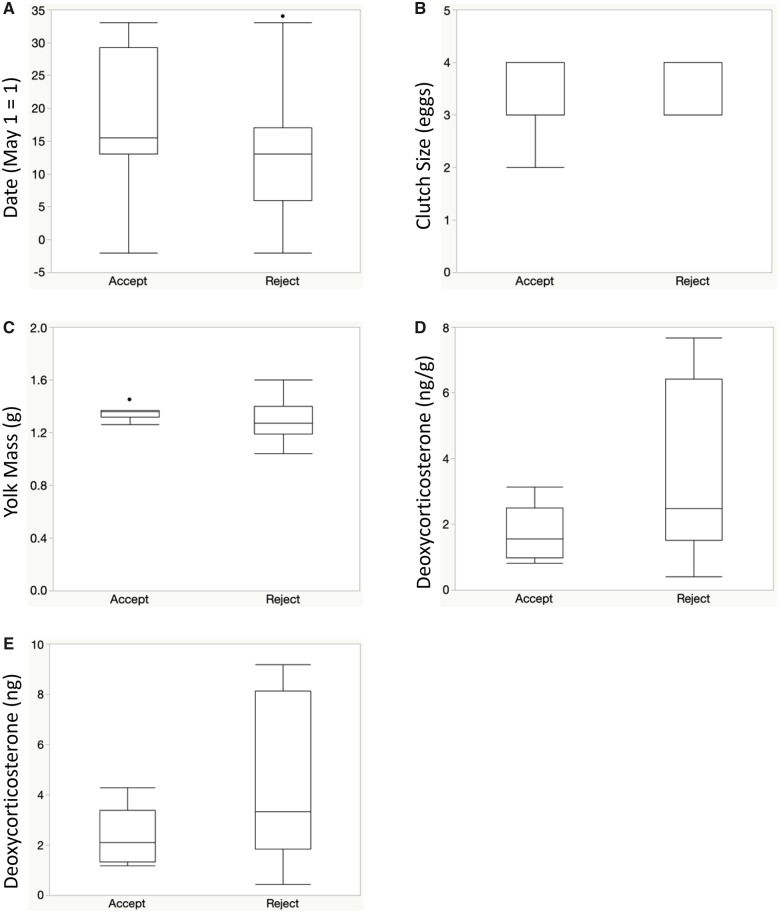
Predictors of maternal investment (**A**) laying date, (**B**) clutch size (# of eggs), (**C**) unicubated yolk mass (g), (**D**) yolk deoxycorticosterone concentration (ng hormone/g), and (**E**) total deoxycorticosterone investment (ng), as a function of the cumulative Day 4 acceptance (*a*; *n* = 8)/rejection (*r*; *n* = 19) decisions (*x*-axis) by American robins. The box plots indicate the 10th, 25th, 50th, 75th, and 90th percentiles and the outliers.

Our nominal logistic regression analyses did not reveal statistically significant relationships between the several proxies of maternal investment into breeding by female robins, including clutch size, laying date, and yolk mass (Table 3, Figure 2A–C). However, when analyzing the first three PC scores of both hormone concentrations and hormone investments, we found that the robins that rejected the model egg by Day 4 had different yolk hormone concentrations and total hormone investment, compared to the accepters (Table 3). Specifically, *post hoc* analyses revealed that both deoxycorticosterone concentration (*R*^2^ = 0.14, χ^2^ = 4.2, *P* = 0.04) and deoxycorticosterone investment (R^2^ = 0.13, χ^2^ = 4.0, *P* = 0.05) were higher in the egg yolk of rejecter individuals relative to accepters (Figure 2D and E).

**Table 3:** Logistic mixed model analysis of Day 4 outcomes (accept/reject) in nests of American robins as predicted separately by several metrics of maternal investment, using site as a random effect

Predictor(s)	*R* ^2^	χ^2^	*P*
Laying date	0.02	0.64	0.43
Clutch size	<0.01	0.02	0.88
Yolk weight	0.04	1.18	0.28
Yolk steroid concentrations	0.23	6.89	
PC1			0.11
PC2			0.084
PC3			0.048
Yolk steroid investment	0.23	6.82	
PC1			0.27
PC2			0.13
** **PC3			**0.029**

Bold values are less than 0.05.

## Discussion

American robins reject most cowbirds eggs when parasitized naturally or experimentally (Rothstein 1982), but a minority accept the dissimilar foreign egg (Lowther 1981), and pay the otherwise recoverable cost of raising a heterospecific young in the brood (Croston and Hauber 2015a). We used a model egg color (deep blue) that is known to induce an intermediate and intra-individually repeatable variation in egg rejection responses of robins (Luro and Hauber 2017), relative to robin’s typically consistently near-100% rejection-responses to natural cowbird eggs and the lack (0%) of the rejection of experimentally-introduced conspecific eggs (Briskie et al. 1992). Regarding the specific context of egg rejection tested by our study, the deep-blue model egg’s avian perceptual distance is 17 Just Noticeable Difference (JND) units relative to the robins’ natural eggs, whereas the natural cowbird egg is ∼14 JND apart from the robin egg and the interclutch discriminability of conspecific robin eggs is ∼3 JND (Croston and Hauber 2014a). Thus, our study design more closely resembled the perceptual task of interspecific rather than intraspecific egg discrimination.

We set out to assess whether an increased maternal investment positively predicts the probably of foreign egg rejection at her nest. We found, at best, weak observational support for this prediction, in that none but one of three overall metrics of maternal investment in the current clutch, and only one of 10 measured steroid hormones correlated with the rejection probability of the model egg. An earlier study at our study site (Abolins-Abols and Hauber 2020), using a comparable sample size (*n* = 31), similarly did not find a relationship between date of treatment across the breeding, but showed that the final clutch size was negatively correlated with egg rejection. However, that earlier study added the deep blue model eggs to robin eggs on the day of or day(s) after clutch completion, which may have substantially lowered the perceived costs of parasitism and, thus, resulted in different relationships between clutch size and egg rejection.

Null results in any correlational study, including ours, may be produced because the sample sizes were too small (but see Abolins-Abols and Hauber 2020 for comparable sample sizes), and/or the proxies did not accurately or sufficiently characterize variation in the extent of the underlying trait(s) (in our case, maternal investment into each breeding attempt). For example, in robins the first egg’s yolk size may only weakly relate to the yolk content and composition produced for the whole clutch (we are planning to assess this in future studies). In turn, variation in some of our proxy metrics may have been too limited (such as in date: our sampling covered a 40-day period only from the onset of the robin breeding season; or in clutch size: most nests contained either three or four eggs; Figure 2B). However, we did find that several yolk hormone concentrations significantly varied over the course of our study, implying biological relevance for our approach to assess date as a seasonal proxy metric for maternal investment (see below). Finally, our different metrics of maternal investment into the clutch did not correlate with each other, except for the seasonal increase in yolk testosterone (as seen in other avian species: Jenni-Eiermann et al. 2020) that was paralleled by decreases in yolk estrone, pregnenolone, and pregnenedione concentrations (Figure 1). However, none of these metrics were again related to patterns of egg rejection behaviors at our focal robin nests. Future studies should assess, therefore, whether the maternal investment mediated foreign egg rejection hypothesis could be more relevant for smaller hosts of cowbirds for which the cost of egg rejection is greater, especially relative to robins that can easily both pierce and grasp reject cowbird eggs (Rasmussen et al. 2009).

The seasonal changes we detected in the PC1 and PC2 endocrine metrics, as well as the temporal changes in individual steroids (Figure 1), were not correlated with variation in egg rejection behavior. Nonetheless, the temporal variation in yolk steroid patterns across the nesting season may have potentially important implications for mediating maternal effects. Accordingly, seasonal variation in egg yolk steroid levels has recently been found in other bird species (e.g., Hargitai et al. 2009; Jenni-Eiermann et al. 2020) and can be positively correlated with seasonal changes in offspring traits, such as growth rates (Jenni-Eiermann et al. 2020). In our study, embryos developing later in the season were likely exposed to lower levels of progestogens and estrogens, but higher levels of androgens (Figure 1). How this variation may mediate seasonal maternal effects on embryonic development in robins remains to be investigated in (our) future studies.

Even in the context of our statistically significant models, where, relative to acceptors, rejecters invested more concentrated deoxycorticosterone in the yolk of the first laid egg, it remains to be assessed whether differential investment of this steroid in fact incurred a differential cost for the laying female (as is known regarding higher yolk androgen deposition, e.g., by older European starlings *Sturnus vulgaris*; Pilz et al. 2003; also see Lessells et al. 2016 for great tits *Parus major*). Alternatively, egg yolk hormone amounts and concentrations may be by-products of maternally circulating endocrine levels, and unrelated to costly and/or strategic maternal investment into the egg yolks and the developing embryos (Williams et al. 2005; Jawor et al. 2007). To evaluate these alternatives will require simultaneous assessment of the laying female’s and her eggs’ yolk hormone levels, perhaps as a function to (perceived) experimental exposure to avian brood parasitism (e.g., Louder et al. 2020; Lawson et al. 2020) .

In our study, we identified differential deoxycorticosterone levels in the egg yolk of acceptors (lower) versus rejectors (higher; Figure 2D and E). This mineralocorticoid is a precursor to corticosterone (Vinson 2011) and can become elevated when corticosterone production is inhibited (Monaghan et al. 2011). However, deoxycorticosterone can also be converted into aldosterone, and the function of aldosterone in avian embryos remains unknown. Whether one or both of these precursory roles of deoxycorticosterone are realized during embryonic development in the avian egg yolk in general, and in robin embryos specifically, also remains unknown.

Finally, despite the statistical patterns reported here, our best models (Table 3) explained no more than 23% of variation in response behaviors of robins to model eggs, with the single-variable model (deoxycorticosterone) explaining no more than 14%. It, therefore, remains to be explored, both observationally and experimentally, what other metrics of maternal investment factors may contribute to the reported acceptance versus rejection responses to the deep blue model egg in individual robins. For example, robins produce immaculate eggs whose shell colors are consistent within a clutch laid by the same individual, but variable between clutches of different individuals (Croston and Hauber 2015b). Therefore, individuals may be more likely accept the deep-blue model egg when it more closely resembles their own eggs’ coloration (i.e., the self-referenced extended phenotype: Hauber et al. 2015). An experimental study may address this scenario by altering the coloration of the robin’s own eggs in the nest, through dyeing or replacing all freshly-laid natural eggs (Strausberger and Rothstein 2009) to generate varying perceptual distances between the model egg from the birds’ “own” eggs (Stevens et al. 2013; Hanley et al. 2017), and assessing the egg rejection rates in response to these manipulated “self” versus foreign color contrasts.

Alternatively, or in addition, robins may be more likely to reject the foreign eggs depending on their own internal phenotype, as seen in other species or predicted in general; for instance, when the breeding female is older and/or more experienced in nesting (Lotem et al. 1992), or when they circulate lower pro-maternal or higher maternally-antagonistic hormone levels (Abolins-Abols and Hauber 2018). In a recent observational study, where female robins were captured, aged, weighed, and assessed for circulating glucocorticoid levels, birds with higher body mass and higher corticosterone plasma concentrations, but not with greater age, were less likely to reject deep blue model eggs (Abolins-Abols and Hauber 2020). Nonetheless, the causal role of these factors still remains to be examined in robins and in most other rejecter host species of avian brood parasites, for example, by manipulating the circulating hormone levels of the incubating females experimentally (Abolins-Abols and Hauber 2019).
